# OLDER ADULTS’ BEHAVIOR IN SEEKING SERVICE INFORMATION DURING THE EARLY COVID-19 PANDEMIC

**DOI:** 10.1093/geroni/igae098.2335

**Published:** 2024-12-31

**Authors:** Leyi Zhou, Cheng Ren, Christine Wei-Mien Lou, Julian Chun-Chung Chow

**Affiliations:** University of California Berkeley, Berkeley, California, United States; University at Albany, SUNY, Albany, New York, United States; San Francisco Human Services Agency, San Francisco, California, United States; University of California Berkeley, Berkeley, California, United States

## Abstract

This study examines the expanding digital divide affecting older adults in the context of the COVID-19 pandemic. It focuses on exploring the factors that influence how older adults access information during COVID-19, emphasizing the challenges faced by those from disadvantaged backgrounds due to limited technology experience. Utilizing survey data conducted in May 2020 from the San Francisco Human Services Agency, which includes responses from 3,255 older adults in households receiving public benefits, this research employs logistic regression analysis to examine how demographic factors—such as race/ethnicity, education level, and primary language—impact the ways in which older adults access information during the pandemic. The analysis encompasses a range of information sources, including both digital and traditional media, to uncover disparities in access. The results unveil stark disparities: Black older adults exhibited significantly lower odds of accessing information via online news and email compared to their White counterparts, indicating a pronounced digital exclusion. Conversely, Chinese older adults demonstrated a higher propensity for social media engagement, suggesting cultural and community-based differences in information dissemination preferences. Education emerged as a critical determinant, with older adults possessing less formal education showing diminished access to digital information sources. These nuanced findings highlight the critical need for targeted interventions aimed at bridging the digital divide. By tailoring educational and training initiatives to the unique needs of diverse older adult populations, the study underscores the importance of ensuring equitable access to essential information, thereby empowering all older adults to navigate public health crises more effectively.

